# Investigation of human cationic antimicrobial protein-18 (hCAP-18), lactoferrin and CD163 as potential biomarkers for ovarian cancer

**DOI:** 10.1186/1757-2215-6-5

**Published:** 2013-01-22

**Authors:** Ratana Lim, Martha Lappas, Clyde Riley, Niels Borregaard, Holger J Moller, Nuzhat Ahmed, Gregory E Rice

**Affiliations:** 1 Department of Obstetrics and Gynaecology, University of Melbourne, Melbourne, VIC, Australia; 2 Translational Proteomics, Baker IDI, Melbourne, Melbourne, VIC, Australia; 3 Mercy Perinatal Research Centre, Mercy Hospital for Women, Heidelberg, VIC, Australia; 4 Women’s Cancer Research Centre, Royal Women’s Hospital, Parkville, VIC, Australia; 5 Department of Hematology, University of Copenhagen, Copenhagen, Denmark; 6 Department of Clinical Biochemistry, Aarhus University Hospital, Aarhus, Denmark; 7 University of Queensland Centre for Clinical Research, Brisbane, QLD, Australia

**Keywords:** Ovarian cancer, hCAP-18, Lactoferrin, CD163

## Abstract

**Background:**

Epithelial ovarian cancer is one of the leading causes of gynaecological cancer morbidity and mortality in women. Early stage ovarian cancer is usually asymptomatic, therefore, is often first diagnosed when it is widely disseminated. Currently available diagnostics lack the requisite sensitivity and specificity to be implemented as community-based screening tests. The identification of additional biomarkers may improve the diagnostic efficiency of multivariate index assays. The aims of this study were to characterise and compare the ovarian tissue immunohistochemical localisation and plasma concentrations of three putative ovarian cancer biomarkers: human cationic antimicrobial protein-18 (hCAP-18); lactoferrin; and CD163 in normal healthy women and women with ovarian cancer.

**Methods:**

In this case–control cohort study, ovarian tissue and blood samples were obtained from 164 women (73 controls, including 28 women with benign pelvic masses; 91 cancer, including 21 women with borderline tumours). Localisation of each antigen within the ovary was assessed by immunohistochemistry and serum concentrations determined by ELISA assays.

**Results:**

Immunoreactive (ir) hCAP-18 and lactoferrin were identified in epithelial cells, while CD163 was predominately localised in stromal cells. Tissue ir CD163 increased significantly (*P*<0.05) with disease grade. Median plasma concentrations of soluble (s)CD163 were significantly greater in the cases (3220 ng/ml) than in controls (2488 ng/ml) (*P*< 0.01). Median plasma concentrations of hCAP-18 and lactoferrin were not significantly different between cases and controls. The classification efficiency of each biomarker (as determined by the area under the receiver operator characteristic curve; AUC) was: 0.67± 0.04; 0.62 ± 0.08 and 0.51 ± 0.07 for sCD163, hCAP-18 and lactoferrin, respectively. When the 3 biomarkers were modelled using stochastic gradient boosted logistic regression, the AUC increased to 0.95 ± 0.03.

**Conclusions:**

The data obtained in this study establishes the localisation and concentrations of CD163, hCAP-18, and lactoferrin in ovarian tumours and peripheral blood. Individually, the 3 biomarkers display only modest diagnostic efficiency as assessed by AUC. When combined in a multivariate index assay, however, diagnostic efficiency increases significantly. As such, the utility of the biomarker panel, as an aid in the diagnosis of cancer in symptomatic women, is worthy of further investigation in a larger phase 2 biomarker trial.

## Background

Each year, more than 200,000 women are diagnosed with ovarian cancer. Ovarian cancer is the sixth most common cancer in women and the second most common type of gynaecological cancer in the world [1]. In the USA, the prevalence of ovarian cancer in postmenopausal women is 1 in 2,500 and the lifetime risk of a woman developing ovarian cancer is 1 in 72. The age-adjusted incidence and death rates for ovarian cancer are 12.7 and 8.2 per 100,000, respectively [2]. The average five-year survival rate for ovarian cancer patients is approximately 46%. This high overall mortality is a consequence of a failure to detect this disease at an early stage. As there are no clinically overt early symptoms, most women (~75%) are first diagnosed with disseminated disease (Stage III/IV) when prognosis is poor. Despite recent progress in chemotherapeutic treatments, the diagnosis of late stage disease is associated with a five-year survival rate of ~20%. In contrast, when ovarian cancer is identified at an early stage, five year survival increases to ~80%. Unfortunately, there is no cost effective screening test currently available [3]. Recent cohort studies [4,5] highlight the failure of the currently available diagnostic tests to identify ovarian cancer early enough to affect disease progression and outcome. Thus, the development of more accurate and earlier detection tests for ovarian cancer in pre-symptomatic women are undoubtedly the number one priority for achieving long-term reduction of mortality from ovarian cancer [3].

Human cationic antimicrobial protein of 18 kDa (hCAP-18) is a major protein of the specific granules of human neutrophils [6]. It is constitutively expressed in a range of inflammatory and epithelial cells, particularly in parts of the body exposed to the outside environment, such as the airway, gut and urinary tract [7,8]; it is also present in squamous epithelia [9] and in keratinocytes during inflammatory skin diseases [10]. hCAP-18 is over-expressed in ovarian cancer tumours and promotes ovarian cancer cell proliferation [11]. Lactoferrin was first recognised as a single-chain iron-binding protein [12-14] and is highly expressed in milk and colostrum [15,16]. Lactoferrin is also found in mucosal secretions and in the specific granules of mature neutrophils [17-19]. Lactoferrin wields broad-spectrum antimicrobial activity against pathogens such as bacteria, fungi, and viruses [17] and is strongly up-regulated during inflammation [20]. It has been demonstrated that lactoferrin displays anti-tumour activity by regulating tumorigenesis [21,22]. Lactoferrin gene polymorphisms have been described in Chinese Han population and were significantly associated with ovarian cancer [23]. CD163 is a haemoglobin scavenger receptor solely expressed in the monocyte-macrophage system and is a mediator against systemic inflammation [24]. A soluble form is present in plasma (sCD163) and has been used as a marker for monocyte/macrophage activity in diseases such as acute myeloid leukaemia [25], rheumatoid arthritis [26,27] and tuberculosis [28]. A number of studies have found an association between CD163 expression and ovarian cancer, notably in the expression of tumour-associated macrophages [29-31].

There is a paucity of data on hCAP-18, lactoferrin and CD163 and their expression in circulating plasma, and in the tumours themselves, from women with ovarian cancer, specifically with differing grades of disease. Thus, in this study, an initial phase 1 biomarker trial (*i.e.* a proof-of-principle, case–control study [32]) was conducted to characterise disease-associated changes in: (i) antigen expression in ovarian tissue (using immunohistochemistry); and (ii) antigen concentration in plasma or serum (determined by ELISA). The diagnostic efficiency of each individual biomarker and in combination as a 3 biomarker panel to correctly identify women with ovarian cancer was established (as assessed by the area under the receiver operator characteristic curve, AUC).

## Methods

### Study design

The experimental design used to evaluate the diagnostic efficiency of plasma concentrations of hCAP-18, lactoferrin and CD163 was a retrospective case control study. Written, informed consent was obtained from patients involved in this study. A total of 164 patients were included in the study. The control group included women with no overt ovarian disease (n=45) and women with benign masses (n= 28). The case cohort included symptomatic women who were subsequently histologically diagnosed with ovarian cancer (n=70). Twenty-one patients were diagnosed with borderline tumours. The study was approved by the Royal Women’s Hospital, Melbourne, Research and Human Ethics Committees (Human Ethics Committee 02/29 and 02/30).

### Blood collection

Whole blood (10 ml) was collected by peripheral venipuncture for serum (blood collection tubes were allowed to clot at room temperature for 30 min) or EDTA tubes for plasma. Samples were centrifuged at 2,000 × *g* for 10 min and the serum or plasma was collected. Samples were stored at −80°C until analysed.

### Tissue collection

To assess whether or not the expression of the three biomarkers was altered in association with the presence of ovarian cancer, where possible, matching ovarian tissue samples were collected from patients. Control tissues were collected from patients undergoing surgery as a result of suspicious ultrasound images, palpable abdominal masses, and from elective oopherectomy. Case tissues were removed at the time of tumour cytoreduction surgery. Histological grading of ovarian carcinoma was performed by the method described by Silverberg [33]. As described previously [34], tissues were snap frozen in liquid nitrogen then stored at −80°C. Resected tissues not required for clinical analysis were obtained from patients who presented for surgery. Serum and plasma samples were collected from patients after diagnosis and before surgery. Where possible, blood and tissue collected from the same patient was used for both IHC and ELISA analysis.

### Immunohistochemistry (IHC)

The expression of hCAP-18, lactoferrin and CD163 in ovarian tissues was also assessed using standard immunohistochemical methodologies. hCAP-18 and lactoferrin immunohistochemical staining was performed using paraffin-embedded tissues as previously described [35]. Rabbit hCAP-18 antibody [6] was used at a concentration of 0.5 mg/ml. A commercially-available lactoferrin antibody (L-3262, Sigma Aldrich, MO, USA) was used at 20 μg/ml. CD163 staining was assessed using frozen sections mounted on poly-l-lysine-coated slides. Frozen tissue sections 5 μm thick were cut at −23°C using a cryostat. Tissue sections were fixed in acetone for 15 min at −20°C then washed in TBS. Endogenous peroxidase activity was blocked with 3% H_2_O_2_ in methanol for 10 minutes. Sections were incubated for 1 h in CD163 antibody (MAC 2–158, Abcam, UK) diluted to 4.5 μg/ml in 1% BSA in TBS. Antibody binding was amplified using biotin and streptavidin HRP for 10 minutes each and the complex was visualised using DAB. Nuclei were stained with Mayer’s haematoxylin. Mouse serum was substituted for the antibody as a negative control.

Additional antibody staining was performed for the CD163 study; macrophages (CD68, diluted to 0.8 μg/ml), endothelial cells (CD31, diluted to 25 μg/ml) and epithelial cells (EMA, diluted to 2 μg/ml) were tested on 2 cases and compared to the CD163 positive stain.

For all IHC analysis except CD163, sections were assessed microscopically for positive DAB staining. The staining was scored blind for the extent of staining. The entire tissue section was scored and the extent of staining was determined on a scale of 0–5 according to the estimated percentage of cells stained: 0, ≤10%; 1, 11–25%; 2, 26–50%; 3, 51–75%;4, 76–90%; 5, ≥90% [36]. For CD163, staining did not seem to be epithelial in nature, so Leica QWin Version 3 Image Analysis Software (Leica Microsystems Imaging Solutions Ltd, Cambridge, UK) was used to quantify tissue staining. Ten random fields of view (FOV) were selected for each slide and for each FOV, a ratio of CD163 stain/whole tissue was determined.

### Enzyme Linked Immunosorbent Assay (ELISA)

Serum and plasma samples were assessed by ELISA as previously described for hCAP-18 [6] and lactoferrin [37]. Serum and plasma samples were assayed for soluble CD163, as described previously [38].

### Statistical analyses

#### Assessing the association between plasma analyte concentrations and disease grade

Multiple group comparisons were assessed by Kruskal-Wallis tests. Dunn’s multiple comparison tests were used for post-hoc two sample comparisons (GraphPad Prism version 5.04 for Windows, GraphPad Software, San Diego, CA, USA). A *P* value of < 0.05 was ascribed as statistically significant. Data are presented as median value and interquartile range.

#### Assessing the association between plasma analyte concentrations in non-malignant and malignant cohorts

Data were partitioned into non-malignant (control = control & benign) and malignant (case = borderline & grade 1–3) groups. Two-sample group comparisons of median values were assessed by Mann Whitney tests (GraphPad Prism). Correlation between two sample groups was assessed by Spearman’s rank correlations using the Bonferroni correction. Data are presented as median value and interquartile range. Statistical significance was assigned at *P* < 0.05.

#### Receiver Operator Characteristic (ROC) curves

Receiver operator characteristic (ROC) curves were used to determine if plasma concentrations of hCAP-18, lactoferrin and sCD163 are of utility in identifying women with ovarian cancer. In addition, the area under the curve (AUC) was used to represent an overall summary of diagnostic performance. A larger AUC indicates better predictability of disease with a value of 1 representing perfect predictive ability. Data were grouped as case (grade 1–3) and control (control and benign) and assessed by ROC analysis using commercially-available software (GraphPad Prism). The AUC was calculated using the Wilcoxon statistic [39]. The diagnostic performance of the individual biomarkers was assessed by comparison of the area under ROC curves using the method of Hanley and McNeil [40] for ROC’s derived from the same cases.

#### Multivariate modelling

Multivariate classification models were developed, based upon observed analyte plasma concentrations using a stochastic gradient boosting model with a logistic loss function as previously described [41] using the WEKA software package [42]. The boosted logistic regression algorithm reported a predicted posterior probability value (*i.e.* the likelihood that a sample came from a woman with ovarian cancer) for each patient sample. Predicted posterior probability values (ppv) were then used to generate ROC curves for each individual analyte and combined in a 3-biomarker panel.

## Results

### Patient characteristics

For the immunohistochemistry studies, a total number of 94 tissue samples were assessed. The mean age of women involved was 57 years, the youngest being 16 years and the oldest 85. For the ELISA assays, a total number of 164 samples were assessed, with the mean age of women being 57 years. Data were divided into histological groups: controls and benign, borderline, grade 1, grade 2 and grade 3 ovarian tumours. Each group of ovarian cancer tissues contained a number of tumour subtypes; mucinous, serous, endometrioid, transitional cell, clear cell, Brenner, or a mixture of two or three subtypes.

### Localisation of hCAP-18, lactoferrin and CD163 in ovarian tissues

#### hCAP-18

**Figure 1:**
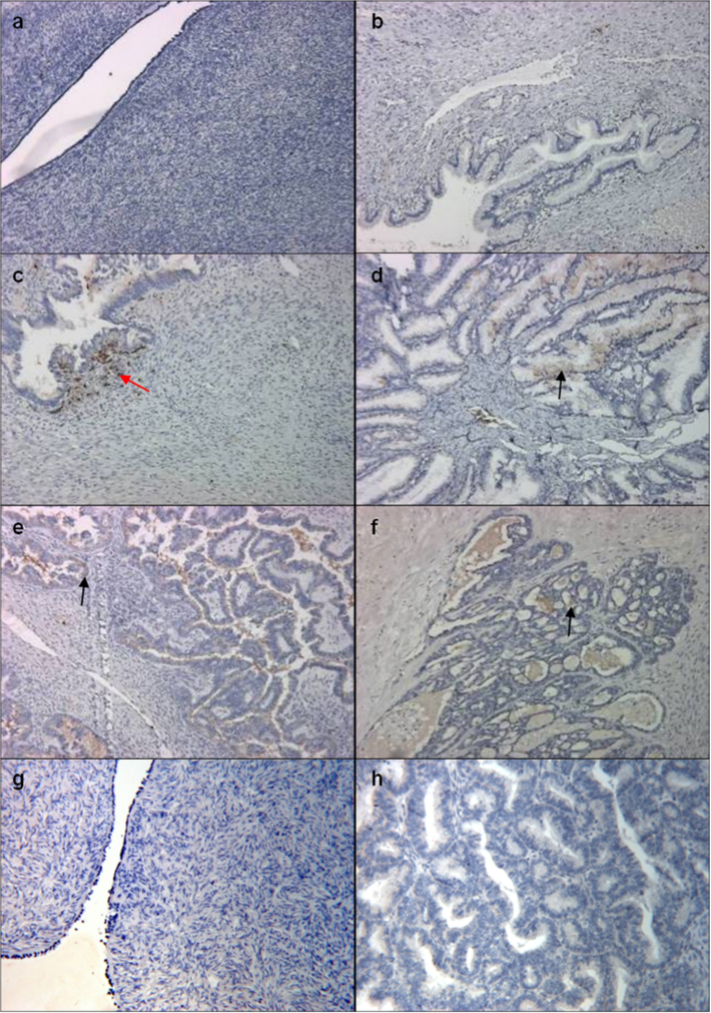
**Immunohistochemical expression of hCAP-18 in ovarian tissues. **Paraffin sections were stained by the immunoperoxidase method as described in Materials and Methods. Representative fields were photographed at 200 X magnification. (**a**) Normal ovarian tissue; (**b**) benign ovarian tissue; (**c**) borderline ovarian cancer tissue; (**d**) grade 1 ovarian tumour; (**e**) grade 2 ovarian tumour; (**f**) grade 3 ovarian tumour. Black arrows denote brown epithelial hCAP-18 staining. Red arrow denotes white cell staining, excluded from extent scores. Negative controls from (**g**) normal and (**h**) grade 1 tumour.

Immunohistochemical localisation of hCAP-18 in epithelial ovarian tumours is demonstrated in Figure 1, for each histological grade. Immunoreactive hCAP-18 was not expressed in normal (n=6, Figure 1a) or benign tissues (n=6, Figure 1b). Some staining was evident in borderline tissues (n=6, average score = 1). Figure 1c shows white cell staining (excluded from the IHC score) in an hCAP-18-negative borderline tissue. Epithelial staining appeared in 4 of the 5 grade 1 tissues (average score = 1.6, Figure 1d). In grade 2 tissues (Figure 1e), 3 of the 6 cases showed epithelial staining (average score = 1). One of six grade 3 tissues (Figure 1f) stained for hCAP-18 (average score = 1). The IHC data of extent of staining for hCAP-18 are represented in Table 1. Only grade 1 tissues were significantly higher than normal tissues (*P* < 0.05). An isotype antibody used as a negative control showed no hCAP-18 staining in normal (Figure 1g) or tumour tissues (Figure 1h).

**Table 1:** Immunohistochemical data of hCAP-18 and lactoferrin in ovarian cancer tissues

**Analyte**	**Control**	**Benign**	**Borderline**	**Grade 1**	**Grade 2**	**Grade 3**
hCAP-18	0.0 ± 0.0 n=6	0.0 ± 0.0 n=6	0.2 ± 0.2 n=6	1.6 ± 0.5 * n=5	1.0 ± 0.5 n=6	0.2 ± 0.2 n=6
lactoferrin	0.3 ± 0.2 n=6	0.0 ± 0.0 n=6	1.3 ± 0.6 n=4	2.4 ± 0.6 * n=5	0.8 ± 0.3 n=4	1.2 ± 0.7 n=5

Immunohistochemical data is presented as mean extent score ± SEM. Statistically significant results compared with control samples are denoted with * (Student’s *t*-test, *P*<0.05).

#### Lactoferrin

**Figure 2:**
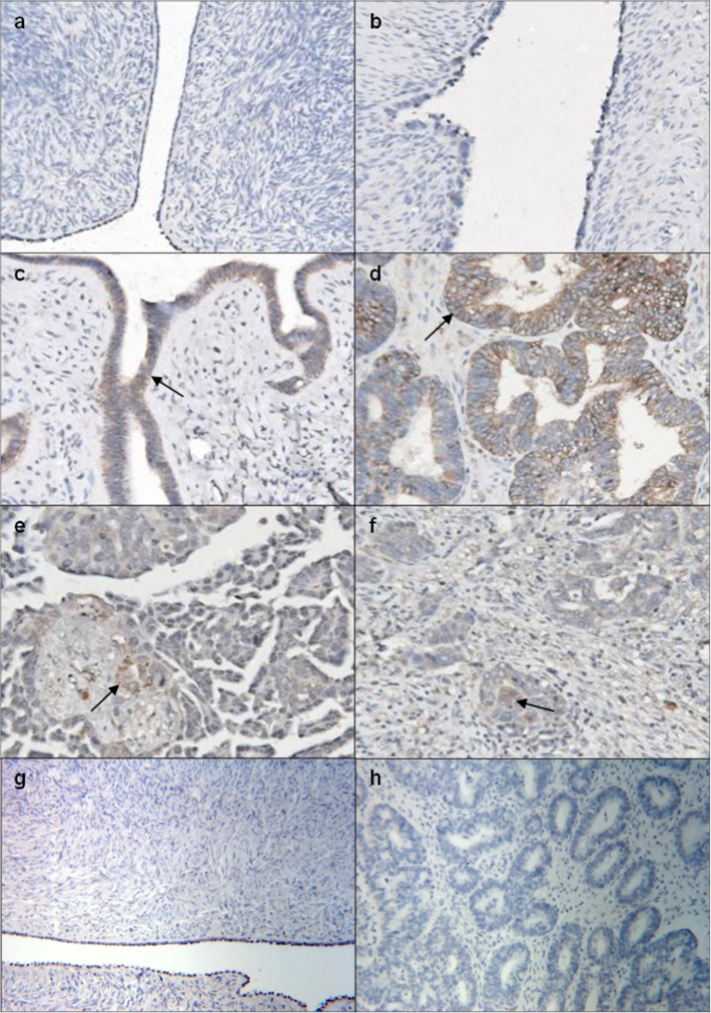
**Immunohistochemical expression of lactoferrin in ovarian tissues.** Paraffin sections were stained by the immunoperoxidase method as described in Materials and Methods section. Representative fields were photographed at 200 X magnification. (**a**) Normal ovarian tissue; (**b**) benign ovarian tissue; (**c**) borderline ovarian cancer tissue; (**d**) grade 1 ovarian tumour; (**e**) grade 2 ovarian tumour; (**f**) grade 3 ovarian tumour. Black arrows denote brown epithelial lactoferrin staining. Negative controls from (**g**) normal and (**h**) grade 1 tumour.

Immunohistochemical expression of lactoferrin in epithelial ovarian tumours is described in Figure 2, which shows a representative image from each histological grade. Figure 2a shows a normal tissue negative for lactoferrin, however two of the 6 cases showed some epithelial staining (extent score = 1). There was no lactoferrin staining in benign tissues (n=6, Figure 2b). Of the borderline tissues (Figure 2c), three of the four cases showed epithelial staining (average score = 1.3). Grade 1 tissues showed the most amount of epithelial staining (n=5, Figure 2d) where all cases showed stain (average score = 2.4). Three grade 2 tissues (Figure 2e) had a score of 1 and one tissue showed no lactoferrin staining. Of the grade 3 tissues, one case had a score of 4 however the remaining 4 cases had a score of 1 or 0, depicted in Figure 2f. An isotype antibody used as a negative control showed no lactoferrin staining in normal (Figure 2g) or tumour tissues (Figure 2h). The immunohistochemical data is represented in Table 1; grade 1 tissues had significantly more lactoferrin stain than control tissues.

#### CD163

**Figure 3:**
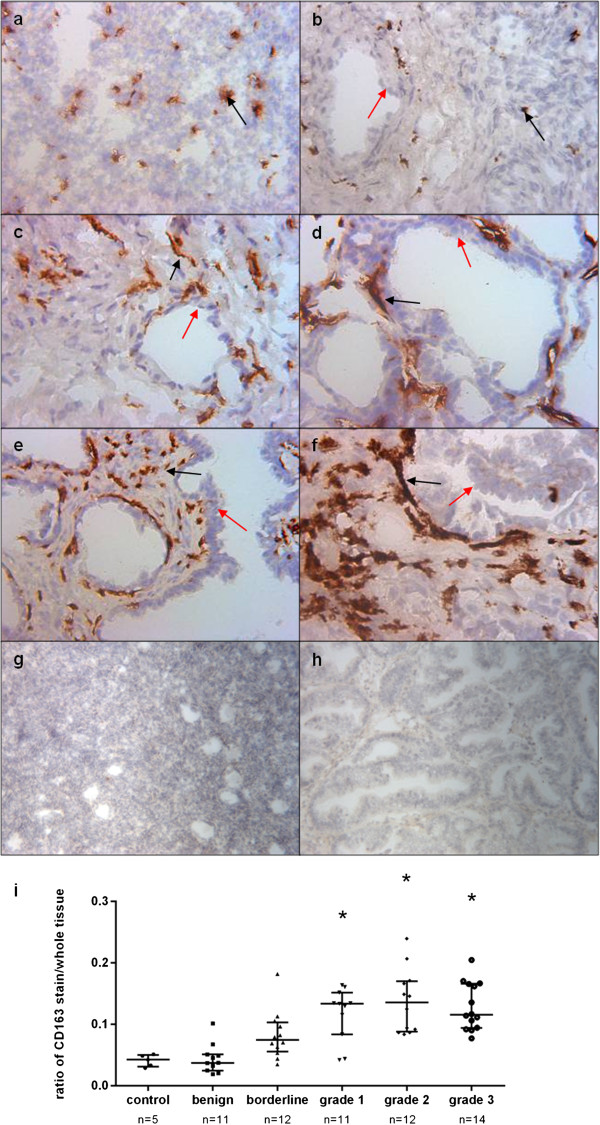
**Immunohistochemical expression of CD163 in ovarian tissues. **Frozen sections were stained by the immunoperoxidase method as described in Materials and Methods. Representative fields were photographed at 200 X magnification. (**a**) Normal ovarian tissue; (**b**) benign ovarian tissue; (**c**) borderline ovarian cancer tissue; (**d**) grade 1 ovarian tumour; (**e**) grade 2 ovarian tumour; (**f**) grade 3 ovarian tumour. Black arrows denote brown CD163 staining in non-epithelial cells. Red arrows indicate unstained epithelial cells. Negative controls from (**g**) normal and (**h**) grade 1 tumour. (**i**) Tissue staining was quantified using Leica QWin Image Analysis Software, determining ratio of stain/whole tissue. Statistically significant results compared with control samples are denoted with * (Mann–Whitney test, *P*<0.05).

A total of 63 frozen tissue samples were used to determine the immunohistochemical expression of CD163 in epithelial ovarian tumours. A representative image from each histological grade is shown in Figure 3a-f. The black arrows show that CD163 staining was confined to the stroma of tissues; no stain was apparent in epithelial cells (red arrows). An isotype antibody used as a negative control showed no CD163 staining in normal (Figure 3g) or tumour tissues (Figure 3h). Using the Leica QWin software, staining was quantitated by creating a ratio of the amount of immunological stain compared to the entire tissue. CD163 staining was evident in all groups of tissues. Grades 1 (n=11), 2 (n=12) and 3 (n=12) ovarian cancers had a significantly higher ratio of stain than normal (n=5) and benign (n=11) tissues (Figure 3i). The amount of CD163 stain increased with tumour grade; staining was found in tumour cells but not of epithelial origin. While the data is not shown, additional antibodies were tested by IHC for endothelial cells (CD31), macrophages (CD68) and epithelial membrane (EMA). Compared to the corresponding CD163 slide, the three antibodies did not match in staining.

### Concentration of hCAP-18, lactoferrin and sCD163 in peripheral blood

#### hCAP-18

**Figure 4:**
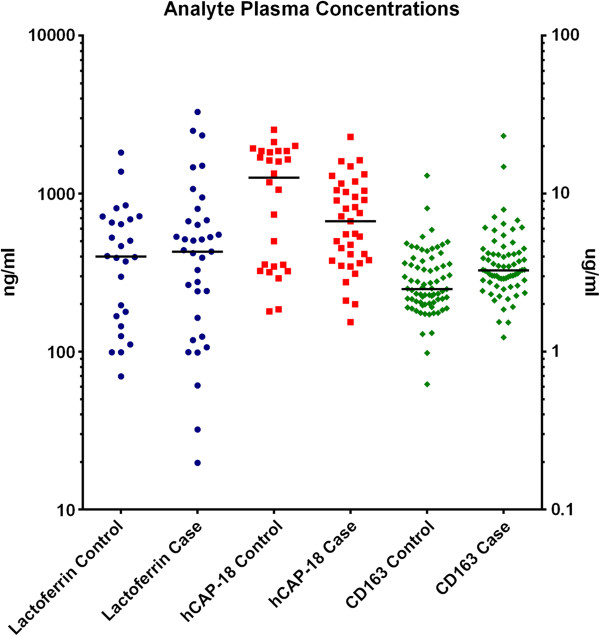
**ELISA analysis of biomarker concentrations in peripheral blood obtained from control and case (ovarian cancer) patients. **Plasma concentrations of lactoferrin (log10 ng/ml, blue circle); hCAP-18 (log ng/ml, orange square), sCD163 (log μg/ml, green diamond) are presented as scatter plots with median bar. Data are stratified by case and control.

Concentrations of hCAP-18 in peripheral blood were measured by ELISA (Figure 4). When data is stratified by histological grade, both benign (n=8) and borderline (n=8) samples were significantly higher than control samples (n=18). Benign samples were 3.9-fold higher, and borderline samples were 5.5-fold higher. No significant differences were found between control samples and tumour samples (n=37) when classified by both grade and stage. When data was stratified by malignancy, *i.e.* non-malignant (control and benign) and malignant (borderline and all graded tumours), there was no significant difference between groups.

### Lactoferrin

Concentrations of lactoferrin in peripheral blood were measured by ELISA (Figure 4). While benign samples (n=8) were 1.5-fold higher than control (n=18), and borderline (n=8) samples were 1.9-fold higher than control, these results were not statistically significant. No differences were detected between the median values of control samples and any of the cancer groups (n=37), nor when data was stratified by malignancy.

#### sCD163

Concentrations of sCD163 in peripheral blood were measured by ELISA (Figure 4c). Data was stratified by histological grade; benign (n=28), borderline (n=21), grade 1 (n=8), grade 2 (n=15) and grade 3 (n=47). No statistical differences were detected between the medians of control samples (n=45) and any of the cancer groups (by grade and stage), nor when data was stratified by malignancy, due to significance in the variance.

#### ROC curve analysis

**Figure 5:**
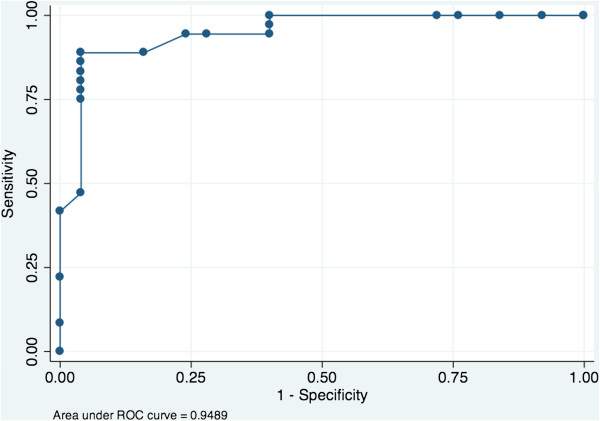
**ROC curve analysis of 3-bimarker model. **Receiver operating characteristic curve based on predicted posterior probability values derived from boosted logistic regression modelling of CD163, hCAP18 and lactoferrin. AUC = 0.949 ± 0.027 (SE).

ROC curves were generated for each analyte to determine their ability to detect ovarian cancer. Each analyte was grouped into control (control and benign) and case (Grade 1–3) samples. Further assessment of data was performed by ROC analysis using commercially-available software. Individually, out of the three biomarkers, sCD163 showed an ability to discriminate between control and ovarian cancer cases, based on raw data (Table 2). When subjected to boosted logistic regression, the posterior probability values (ppv) generated for each biomarker showed improved classification performance, as assessed by increased AUC (Table 2 and Figure 5).

**Table 2:** Comparison of the AUC of ROC curves for individual and combined analytes

**Analyte**	**AUC (raw data)**	**AUC (modelled data)**
CD163	0.67 ± 0.04 *	0.79 ± 0.04 *
hCAP18	0.62 ± 0.08	0.89 ± 0.04 *
Lactoferrin	0.51 ± 0.07	0.80 ± 0.06 *
**3-biomarker panel**	**-**	0.95 ± 0.03 *

Statistically significant results compared with control samples are denoted with * (Student’s *t*-test, *P*<0.0005).

## Discussion

The aim of this study was to establish the immunohistochemical localisation of 3 putative biomarkers of ovarian cancer and evaluate their utility as aids in the diagnosis of ovarian cancer in symptomatic women.

The data obtained identify cancer-associated changes in the immunohistochemical expression of all 3 antigens within the ovary. Immunoreactive hCAP-18 and ir lactoferrin was localised to ovarian epithelial cells and its expression was greater in cases compared to controls. The expression of ir CD163 was confined to stromal cells but was similarly increased in tissues obtained from women with ovarian cancer. The ir expression of all 3 biomarkers was significantly increased in grade 1 ovarian cancer tissues. These data are consistent with the hypothesis that these mediators may participate in cell transformation and development of metastatic potential.

Individually, the plasma concentrations of the biomarkers displayed only modest diagnostic efficiency (as indicated by AUC of less than 0.7). In combination as a 3-biomarker panel (multivariate index assay, MIA), however, AUC increased to 0.95 and, as such, may be of utility as an aid in the diagnosis of ovarian cancer in symptomatic women. While this phase 1 biomarker trial provides an estimate of diagnostic efficiency based on AUC, a larger phase 2 biomarker trial would be required to provide robust estimates of the sensitivity and specificity of the MIA.

Previously, hCAP-18 has been described in breast cancer [43], where hCAP-18 was constitutively expressed in normal mammary gland epithelium and significantly increased in high-grade tumours. While there was some variance of hCAP-18 expression within groups, the study concluded that there was a potential correlation between degree of malignancy of breast cancer and hCAP-18 expression. This association was further explored [44], finding that treatment with hCAP-18/LL-37 altered the growth phenotype of breast cancer cells and stimulated migration. Together with a lung cancer study [45] that also found over-expression of hCAP-18/LL-37 increased tumour growth, it is concluded that hCAP-18 contributes to cancer metastasis.

Differential expression of hCAP-18 has also been reported in ovarian cancer [11], where it was over-expressed in ovarian cancer tumours when compared to normal ovarian tissue. This finding correlates with the breast and lung cancer studies, where high-grade tumours express elevated levels of hCAP-18 when compared to controls. Our study, however, shows that normal ovarian tissue does not express hCAP-18 and the highest amount of staining was in grade 1 tumour, not grade 3. The concentration of hCAP-18 in blood was significantly increased in benign and borderline samples when compared to controls and graded tumours. This dissimilarity may be due to a number of factors; very limited sample numbers, or there could be a difference in the expression of soluble hCAP-18 in the blood and expression in tissues. There are, however, no studies that measure circulating hCAP-18 concentrations in the blood of cancer patients to verify this difference. Besides the recent studies of its role in cancer metastasis, hCAP-18 has been mostly been linked with wound healing and inflammatory disorders [10,46,47]. The results found in this study indicate the need for further studies using a larger cohort of blood samples.

CD163 has been identified, previously, in non-neoplastic monocytes/macrophages and neoplasms of monocyte/histiocyte derivation [48]. CD163 is strongly expressed in “tumour-associated macrophages” and in a number of cancer types its expression is associated with survival [49,50], reflecting the tumour supportive nature of tumour-associated macrophages [51]. Monocytes express CD163 constitutively at low levels, and expression increases during macrophage differentiation [52] and infiltration [53]. Increased sCD163 concentrations in plasma have been reported in pathological conditions, including sepsis and liver disease [54]. There, however, is no clinical or biochemical evidence for inflammatory co-morbidity that explains the increase in sCD163 concentrations in these patients. It, therefore, is possible that the increased sCD163 is directly related to tumour-associated macrophages and other bone marrow-derived cells involved in *e.g.* tumour angiogenesis [51].

The present study involves analysis of three biomarkers in women with ovarian cancer, ranging from normal and benign to all grades of disease. While analysis of each group separates this study from others in the literature, the small sample sizes is a limitation and also the crux of the problem, as ovarian cancer is rarely caught in its early stages. Further, the biomarkers should be compared to CA-125, the gold standard in detecting advanced ovarian cancer; however, CA-125 was not measured in the control group (including benign and borderline samples) and analysis cannot be performed. While age did not affect the results in this study (data not shown), of note is whether patient history of inflammatory diseases and tumour subtype affected the expression of each biomarker. These cannot be addressed for this study (inability to access patient history after project conclusion and very small sample size), but future studies building tissue banks would benefit from obtaining these data sets for their samples.

## Concluding comments

The data obtained in the study define ovarian cancer-associated changes in the immunohistochemical expression and plasma concentrations of three putative biomarkers. When the biomarkers are combined as a multivariate index assay, a diagnostic efficiency was achieved that is commensurate with utility as an aid in the diagnosis of ovarian cancer in symptomatic women. Interpretation and application of the data obtained in this study must include a number of caveats, including: the retrospective case–control design; and small cohort size. A phase 2 biomarker trial would be required to address these caveats and provide robust estimates to the sensitivity and specificity of the multi-marker panel.

## Abbreviations

hCAP-18: human cationic antimicrobial protein-18; IHC: Immunohistochemistry; AUC: Area under the curve; ppv: Positive predictive value; IVDMs: *In vitro *diagnostic multivariate index assays.

## Competing interests

The authors declare that they have no competing interests.

## Authors’ contributions

RL was responsible for study design, performing IHC, collation of all raw data, statistical analysis, data interpretation and writing of the manuscript. GER and NA were responsible for overall study conception, obtaining human ethics approvals and contributed to critical review of the manuscript. CB contributed to IHC and data analysis of IHC. NB and HJM contributed to ELISA data acquisition and manuscript review. ML contributed to data interpretation and critical manuscript review. All authors reviewed the data presented, its analysis and interpretation, and approved the final manuscript.
